# Factors underpinning the performance of implemented artificial intelligence-based patient deterioration prediction systems: reasons for selection and implications for hospitals and researchers

**DOI:** 10.1093/jamia/ocae321

**Published:** 2025-01-16

**Authors:** Anton H van der Vegt, Victoria Campbell, Shuyi Wang, James Malycha, Ian A Scott

**Affiliations:** Centre for Health Services Research, The University of Queensland, Brisbane, QLD 4102, Australia; Intensive Care Unit, Sunshine Coast Hospital and Health Service, Birtinya, QLD 4575, Australia; Patient Safety and Quality, Clinical Excellence Queensland, Brisbane, QLD 4001, Australia; School of Medicine, Griffith University, Sunshine Coast University Hospital, Birtinya, QLD 4575, Australia; School of Electrical Engineering and Computer Science, The University of Queensland, St Lucia, QLD 4067, Australia; Intensive Care Department, Royal Adelaide Hospital, Adelaide, SA 5000, Australia; Centre for Health Services Research, The University of Queensland, Brisbane, QLD 4102, Australia; Metro South Digital Health and Informatics, Princess Alexandra Hospital, Brisbane, QLD 4102, Australia

**Keywords:** clinical deterioration prediction, early warning tool, health informatics, digital health

## Abstract

**Objective:**

The degree to which deployed artificial intelligence-based deterioration prediction algorithms (AI-DPA) differ in their development, the reasons for these differences, and how this may impact their performance remains unclear. Our primary objective was to identify design factors and associated decisions related to the development of AI-DPA and highlight deficits that require further research.

**Materials and Methods:**

Based on a systematic review of 14 deployed AI-DPA and an updated systematic search, we identified studies of 12 eligible AI-DPA from which data were extracted independently by 2 investigators on all design factors, decisions, and justifications pertaining to 6 machine learning development stages: (1) model requirements, (2) data collection, (3) data cleaning, (4) data labeling, (5) feature engineering, and (6) model training.

**Results:**

We found 13 design factors and 315 decision alternatives likely to impact AI-DPA performance, all of which varied, together with their rationales, between all included AI-DPA. Variable selection, data imputation methods, training data exclusions, training sample definitions, length of lookback periods, and definition of outcome labels were key design factors accounting for most variation. In justifying decisions, most studies made no reference to prior research or compared with other state-of-the-art algorithms.

**Discussion:**

Algorithm design decisions regarding factors impacting AI-DPA performance have little supporting evidence, are inconsistent, do not learn from prior work, and lack reference standards.

**Conclusion:**

Several deficits in AI-DPA development that prevent implementers selecting the most accurate algorithm have been identified, and future research needs to address these deficits as a priority.

## Background and significance 

Clinical deterioration in hospitals is defined as “an acute worsening of a patient’s clinical status that poses a substantial increase to an individual’s short-term risk of death or serious harm.”^1^ This affects approximately 2% of US hospital inpatient admissions and can result in death or serious morbidity.^2^^,^^3^ Artificial intelligence-based deterioration prediction algorithms (AI-DPA) using supervised machine learning (ML) can identify deteriorating patients with greater accuracy than existing rule-based methods^4–6^ such as the National Early Warning Score (NEWS).^7^ However, AI-based clinical systems bring added complexity as well as costs and implementation challenges,^8–11^ which require AI-DPA predictive accuracy to be sufficiently superior to justify its use over simpler, cheaper, and readily available rule-based tools.

Most AI-DPA evaluation studies are observational and retrospective and few describe systems deployed into clinical practice.^12–14^ A recent review found 14 deployed AI-DPA,^3^ of which 12 provided retrospective analyses and 1 reported post-implementation accuracy.^15^ Only 1 study compared the accuracy of their algorithm with any other deployed AI-DPA.^16^

Meaningful AI-DPA comparisons are hindered by heterogeneity in model development, including the use of different datasets, algorithms, variables, outcomes, evaluation methodologies, and model replication details. This heterogeneity and lack of like-for-like comparisons limit our understanding of the most important design decisions in AI-DPA development, such as which variables generate the most accurate models.

### Objective

The primary objective of this study was to de-construct deployed AI-DPA development and to identify and account for different design decisions that can impact AI-DPA accuracy at each stage in the following standard machine learning workflow^17^: (1) model requirement, (2) data collection, (3) data cleaning, (4) data labeling, (5) feature engineering, (6) model training, and (7) model evaluation, model deployment. Using these findings, the secondary objective was to identify potential areas of research that may enable future AI-DPA development teams to make consistent, transparent, and translatable design decisions for improving accuracy.

## Materials and methods

### Identification of deployed AI-driven early warning systems

Implemented AI-DPAs were identified in 2 stages. First, all systems identified (n = 14) in a recent systematic review^3^ of implemented AI-DPAs were screened for sufficient development detail for data extraction, ie, at a minimum, the studies (or previously cited studies) reported the algorithm, variables, and outcomes used for model development. Second, to ensure we included the latest systems, we conducted a top-up systematic search from the prior review search end date (April 1, 2023) to November 13, 2024, following the same methods previously used by our group.^3^ In brief, 4 databases (PubMed/MEDLINE, EMBASE, Scopus, and Web of Science) were searched for titles and abstracts published in English using keywords and synonyms for (1) predict; AND (2) clinical deterioration; AND (3) machine learning; AND (4) trial; and NOT (5) child. Studies were screened independently by 2 authors (A.H.V. and S.W.) and full text reviews excluded studies that were duplicates or failed to meet the aforementioned eligibility criteria.

### Data extraction

In first identifying major AI-DPA development steps and a draft set of design factors impacting accuracy, we started with the highly acknowledged standard practice 9-stage ML workflow of Amershi et al^17^: (1) model requirement, (2) data collection, (3) cleaning, (4) labeling, (5) feature engineering, (6) model training, (7) evaluation, (8) deployment, and (9) monitoring. We selected the first 6 development stages and constructed an Excel template into which 2 data scientists (A.H.V. and S.W.) independently extracted design factors (ie, specific model construction tasks that could impact model accuracy) from the first 2 randomly selected AI-DPA studies, inserting each factor beneath the relevant pathway stage (see Table 1). The data scientists then harmonized their results and agreed naming conventions prior to completing data extraction for all included studies. A final validation check was done by mapping items in the TRIPOD+AI reporting guidelines^18^ (Table 2) to the identified design factors (see Table 1) to ensure no relevant reporting guideline items were missing. In addition to design factors, data extraction also included the specific design decisions (herein referred to as alternatives) and their justifications, when reported. If, during extraction, a new design factor or decision alternative was found, these were named and added to the template. Upon completion of data extraction, both extractors compared results and discrepancies were resolved by consensus.

**Table 1.** Mapping of Amershi et al,^17^ machine learning pathway stages to design factors identified through coding process of eligible studies to TRIPOD+AI reporting items.

**Machine learning stage** ^17^ (Figure 1)	Coded design factors identified from studies	**Matching TRIPOD+AI reporting guideline item** ^18^ (Table 2)
1. Model requirements	• [1.1] Algorithm selection	• Item 12c: specify the type of model
2. Data collection	• [2.1] Variable selection	• Items 9a: describe the choice of initial predictors • Item 9b: define all predictors
3. Data cleaning	• [3.1] Data exclusions • [3.2] Data error handling • [3.3] Missing value imputation	• Item 7: data pre-processing and quality checks • Item 11: how missing data are handled
4. Data labeling	• [4.1] Outcome selection • [4.2] Positive sample horizon to event, ie, the number of hours preceding an outcome within which a sample has a positive deterioration label	• Item 8a: define the outcome that is being predicted and the time horizon
5. Feature engineering	• [5.1] Training sample definition, eg, discrete or bundled (aggregated) • [5.2] Data lookback context, ie, how far back to look for a variable value if none currently exists • [5.3] Aggregations in lookback context • [5.4] Final data transformation	• Item 12b: describe how predictors were handled in the analyses (functional form, rescaling, transformation, and standardization)
6. Model training	• [6.1] Hyper-parameter optimization • [6.2] Class sampling technique, such as over/under sampling	• Item 12c: all model building steps including any hyperparameter tuning

**Table 2.** Count of algorithm (decision factor 1.1) decision alternatives selected by each deployed AI-DPA and identification of which AI-DPA systems provided explicit justifications for their selection.

**Stage 1: Model requirements** *Design factor* and decision alternatives	DI^19^	Duke Model 1^20^	DEWS^21^	APPROVE^22^	eCART^4^	MEWS++^5^	AAM^16^	Duke Model 2^23^	CHARTwatch^24^	MC-EWS^25^	CONCERN^26^^,^^27^	eCARTv5^28^	Total
*1.1 Algorithms*													
Logistic regression	1				1		1	1	1		1		6
Random forest				1		1							2
Gradient boosting machine		1								1	1	1	4
Multi-variate adaptive regression spline									1				1
Long-short-term-memory			1										1
Ensemble									1		1		2
**Decision justifications** ^a^		**1**	**1**			**1**	**1**	**1**	**1**	**1**		**1**	**8**
Comparative model testing		1	1			1	1		1	1			6
Algorithm characteristics			1			1			1	1		1	5
Less complex to operate		1					1	1					3
Provides transparency						1	1						2

a Bold indicates a decision justification exists for that AI-DPA system.

## Results

### Eligible AI-DPAs

**Figure 1.**
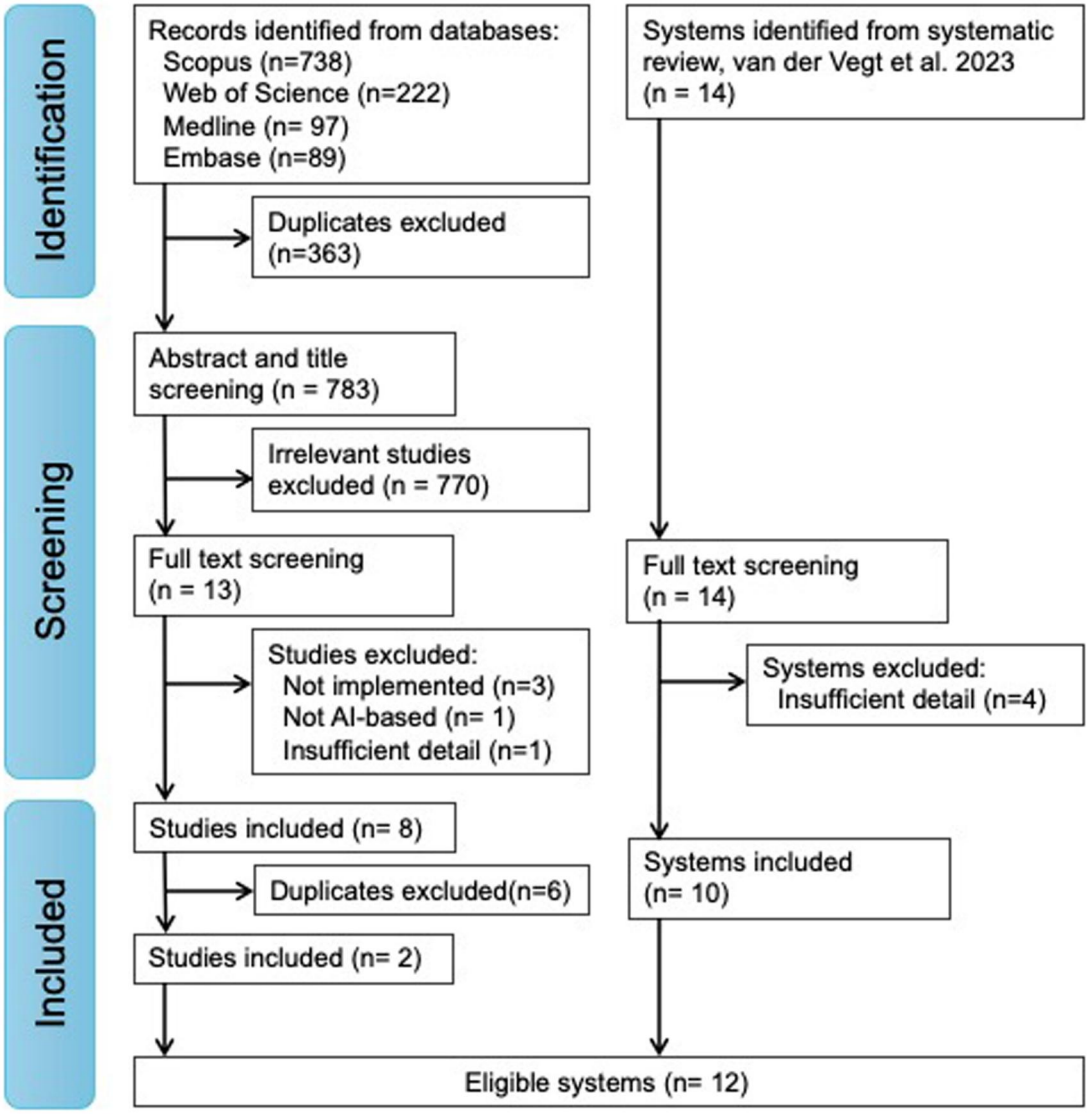
PRISMA flow diagram of AI-DPA search process.

Referring to Figure 1, of the 14 deployed AI-DPA identified within the recent systematic review,^3^ 3 provided no retrospective development details^29–31^ and 1 provided limited information (no variable data) and did not respond to email requests; all 4 were excluded,^15^ leaving 10 AI-DPA for analysis.

In the top-up systematic search, we identified 783 studies from screening, 13 underwent full text review of which 4 were not implemented or not AI-based and one, the CoMET system,^32^^,^^33^ had insufficient detail for analysis. This left 8 studies of which 6 were associated with systems already included from the prior systematic review, resulting in 2 new systems that yielded a total of 12 AI-DPA for analysis: Deterioration Index (DI)^19^; Duke Model 1^20^; Deep Learning-Based Early Warning System (DEWS)^21^; Accurate Prediction of Prolonged Ventilation (APPROVE)^22^; electronic Cardiac Arrest Risk Triage (eCART)^4^; Modified Early Warning Score (MEWS++)^5^; Advanced Alert Monitor (AAM)^16^; Duke Model 2^23^; CHARTwatch^24^; Mayo Clinic Early Warning Score (MC-EWS)^25^; COmmunicating Narrative Concerns Entered by RNs (CONCERN)^26^^,^^27^; and eCARTv5.^28^

### Design factors

**Figure 2.**
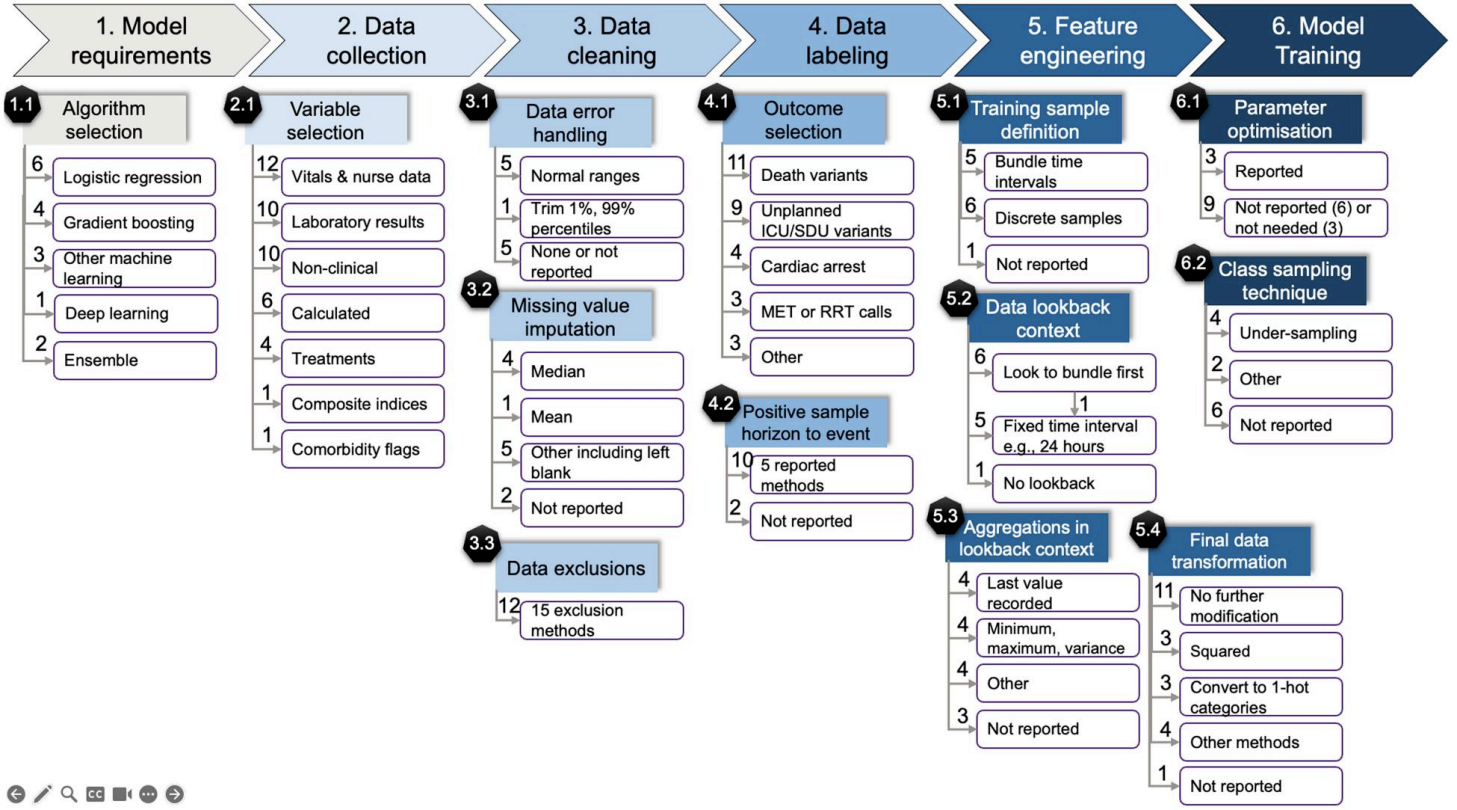
Summary diagram showing numbered Amershi et al.^17^ ML model development stages (1-6, shaded arrows), design factors (1.1-6.2 black pentagon earmarked boxes) that could impact AI-DPA accuracy found at each development stage and the summary-level decision alternatives (in white) together with a count of AI-DPA deciding on a particular alternative included within each summary-level item. Note that AI-DPAs could utilize multiple decision alternatives within each design factor, eg, multiple training label outcomes (4.1). Abbreviations: MET: medical emergency team; RRT: rapid response team; ICU: intensive care unit; SDU: step down unit; ML: machine learning.

We found 13 design factors that could impact AI-DPA accuracy across the 6 AI-DPA development steps defined by Amershi et al.^17^ All are depicted in Figure 2, with development steps in shaded arrows, design factors in shaded boxes, and summary-level decision alternatives in white boxes. The results for each stage are reported below.

### AI-DPA development stage 1: model requirements

Half of the deployed AI-DPAs used logistic regression algorithms (see Table 2),^4^^,^^16^^,^^19^^,^^23^^,^^24^ DEWS used a long-short-term-memory deep learning model,^21^ and the remaining 5 used a variant of decision tree classifiers, including random forest^5^^,^^22^ and gradient boosting machines.^20^^,^^25^^,^^26^^,^^28^ Only CHARTwatch and CONCERN used an ensemble methodology with multiple algorithms.^24^^,^^26^

Eight AI-DPA described at least one justification for algorithm selection. Half the studies selected algorithms based on comparative testing with other algorithms^5^^,^^20^^,^^21^^,^^24^^,^^25^; 5 studies described inherent algorithm benefits, such as ability to handle missing data or non-linear data^5^^,^^21^^,^^24^^,^^25^^,^^28^; 3 described operational limitations to using more complex algorithms, eg, utilizing EPIC’s inbuilt logistic regression setup^5^^,^^16^^,^^20^; 2 described algorithm transparency, such as feature importance^5^^,^^25^; and 4 did not describe any reason.^4^^,^^19^^,^^22^^,^^26^

### AI-DPA development stage 2: data collection

The deployed AI-DPAs used a median of 36 variables (range 4-111) totaling 226 distinct variables (see Table 3), of which 71 were vital sign and nurse captured data used by all AI-DPAs (median = 10.5, range 4-35), 101 were laboratory results used by 10 AI-DPA (median = 17.5, range 0-91), 20 were non-clinical variables used by 10 AI-DPA (median = 4, range 0-8), 18 were calculated variables used by 6 AI-DPA (median 1, range 0-10), 13 were treatment variables used by 4 AI-DPA (median = 0, range 0-10), 2 were composite indices used by AAM^16^ only, and 8 were comorbidity flags used by 1 AI-DPA.^23^

**Table 3.** Count of variables (design factor 2.1) used within each deployed AI-DPA.

**Stage 2: Data collection** (design factor 2.1) *Variable domains* and decision alternatives for each	DI^19^	Duke Model 1^25^	DEWS^21^	APPROVE^22^	eCART^4^	MEWS++^5^	AAM^16^	Duke Model 2^23^	CHARTwatch ^24^	MC-EWS^25^	CONCERN^26^^,^^27^	eCARTv5^28^	Total
*Vitals and nurse captured data*													
Systolic blood pressure	1	1	1	1	1	1	1	1	1	1		1	11
Heart rate/pulse rate	1	1	1	1	1	1	1	1	1	1		1	11
Respiratory rate	1	1	1	1	1	1	1	1	1	1		1	11
Body temperature	1	1	1	1	1	1	1	1	1	1		1	11
Diastolic blood pressure	1	1		1	1	1	1	1	1	1		1	10
Oxygen saturation	1	1		1	1	1	1	1	1	1		1	10
State of consciousness (AVPU)	1				1	1		1				1	5
Supplemental oxygen		1		1				1		1			4
Other 2-off variables		2		1			1		1	6		5	16
Other 1-off variables						5			5	5	35	5	55
Sub-total vitals count	7	9	4	8	7	12	7	8	12	18	35	17	144
Sub-total unique vital count													71
*Laboratory results*													
Blood urea nitrogen/Urea	1	1		1	1	1	1	1	1	1		1	10
White blood cell count	1	1		1	1	1	1	1	1	1		1	10
Sodium		1		1	1	1	1	1	1	1		1	9
Creatinine		1		1	1	1	1		1	1		1	8
Total bilirubin		1		1	1	1		1	1	1		1	8
Potassium		1		1	1	1		1	1	1		1	8
Bicarbonate				1	1	1	1		1	1		1	7
Anion gap				1	1		1	1	1	1		1	7
Glucose		1		1	1		1		1	1		1	7
Lactate (arterial blood gas)		1		1		1	1	1	1	1			7
Albumin		1		1	1			1	1	1		1	7
Hemoglobin	1			1	1	1			1	1		1	7
Platelet		1			1	1		1	1	1		1	7
Hematocrit		1		1		1	1	1	1				6
Aspartate aminotransferase (AST)		1			1			1	1	1		1	6
International normalized ratio (INR)		1				1		1	1	1		1	6
Partial pressure oxygen		1		1				1	1	1		1	6
Troponin		1					1	1	1	1			5
Partial pressure CO_2_ (arterial)		1		1				1	1			1	5
Magnesium		1						1	1	1		1	5
pH (acidity) test arterial		1		1				1	1			1	5
Calcium				1	1				1	1		1	5
Lactate (venous blood gas)				1		1		1	1	1			5
Alanine aminotransferase (ALT)		1						1	1	1			4
Partial pressure CO_2_ (venous)		1						1	1			1	4
Alkaline phosphatase (ALP)					1				1	1		1	4
Neutrophils (also bands)		1							1	1		1	4
C-reactive protein		1						1	1	1			4
Erythrocyte sedimentation rate (ESR)		1						1	1	1			4
pH (acidity) test—venous		1						1	1			1	4
Chloride				1		1			1			1	4
Ammonia		1						1		1			3
Estimated glomerular filtration rate	1					1				1			3
Creatine kinase		1						1	1				3
Lactate dehydrogenase (LDH)		1						1	1				3
Activated partial thomboplastin time									1	1		1	3
Lipase									1	1		1	3
Phosphorus									1	1		1	3
Total protein (albumin + globulin)					1				1			1	3
Other 2-off variables		6				1		5	12	4		6	34
Other 1-off variables									41	2		1	44
Sub-total labs count	4	33	0	19	16	16	10	31	91	36	0	35	244
Sub-total unique labs count													101
*Non-clinical variables*													
Age	1	1		1	1	1	1	1	1			1	9
Gender	1	1		1			1	1	1	1			7
Transpired length of stay					1	1	1	1	1	1		1	7
Weight		1		1		1				1			4
Admission type		1				1	1						3
Height				1		1				1			3
Other 2-off variables	1	2				2		1		2			8
Other 1-off variables					1	1	4			2		2	10
Sub-total non-clinical count	3	6	0	4	3	8	8	4	3	8	0	4	51
Sub-total unique non-clinic count													20
*Calculated variables*													
Shock index (heart rate/systolic bp)				1			1			1			3
Bun creatinine ratio (BUN/Creatinine)				1	1					1			3
Other 1- or 2-off variables				2	1		2		1	12		1	19
Sub-total calculated count	0	0	0	4	2	0	3	0	1	10	0	1	23
Sub-total unique calculated count													18
*Treatments*													
Total IV fluids	1	1							1				3
Other 1- or 2-off variables		9		1					3				13
Sub-total treatment count	1	10	0	1	0	0	0	0	4	0	0	0	16
Sub-total treatment count													13
*Composite indices*													
Other 1-off variables							2						2
Sub-total index (+ unique) count:	0	0	0	0	0	0	2	0	0	0	0	0	2
*Comorbidity flag variables*													
Other 1-off variables								8					8
Sub-total comorbidity count	0	0	0	0	0	0	0	8	0	0	0	0	8
Grand total unique variable count	15	58	4	36	28	36	30	51	111	74	35	57	226
**Decision justifications** ^a^	**1**	**1**	**1**	**1**	**1**	**1**	**1**	**1**			**1**	**1**	**10**
Variable selection experiments	1				1	1	1				1		4
Variable availability/accessibility		1	1	1	1			1					5
Prior work												1	1

All variables that are used by 3 or more AI-DPA are itemized by variable domain, with 1-off and 2-off variables counted in the table, but not listed (see Appendix S1 for full listing). Variables are listed in descending order of use, as provided in the right-hand column and total is provided for each variable domain and overall. Explicit decision justifications are provided at the bottom.

a Bold 1 indicates the system has a decision justification and the specific justification is itemised below.

In terms of overlap, CONCERN was an outlier with variables based wholly on nurse captured data. Excluding CONCERN, 46% (n = 87) of variables were used by more than one AI-DPA with 14% (n = 26) used by more than half. Only 4 variables (systolic blood pressure, heart rate, respiratory rate, and body temperature) were common to all AI-DPAs (excluding CONCERN) and further 6 variables (diastolic blood pressure, oxygen saturation, sodium, white blood cell count, blood urea nitrogen, and age) were common to at least 9 AI-DPA.

In terms of documented decisions for variable selection, all but 2 AI-DPAs^24^^,^^25^ reported justifications, the highest frequency of reporting for all design factors, with 5 selecting variables commonly collected in the electronic medical record (EMR)^4^^,^^20–23^; 4 using experimental selection processes, such as feature reduction^4^^,^^5^^,^^16^^,^^19^; and eCARTv5 referring to prior work.^28^ No AI-DPA explained selection of their initial candidate variables nor whether clinician input was considered.

### AI-DPA development stage 3: data cleaning

We found data was cleaned in 3 ways:

- Data errors, such as implausible vital signs, were removed. Half the AI-DPA did not report or did not perform data error handling; of those which did (Table 4), most (n=5) specified plausible vital sign ranges.^16^^,^^21^^,^^23^^,^^25^^,^^28^ No justifications were provided for the method selected except for eCARTv5.^28^
- For algorithms, including logistic regression algorithms, requiring a full complement of numerical input variable values, imputation methods must be used to account for missing values, with median value imputation (ie, using the training set median of the missing variable) being most commonly used (n=3)^4^^,^^5^^,^^21^ out of 6 methods reported. Reasons for selecting particular imputation methods were reported in 4 AI-DPA, 3 based on prior work^4^^,^^5^^,^^22^ and 1 to suit their algorithm selection.^20^
- Data may be excluded from the training dataset to improve the training process. We found 9 AI-DPA which selected adult patients in wards and excluded ICU and surgery units. Twelve other specific exclusions were identified: 5 to limit specific patient admissions, such as maternity, psychiatric, or palliative admissions; 3 were outcomes-based exclusions, such as outcomes occurring within 30 minutes of admission; and 3 excluded admissions longer or shorter than specified. The same exclusion was used by at-most 3 AI-DPA. Justifications for exclusions were provided by 4 AI-DPA, for example, to reduce training bias^16^^,^^24^ for admission length exclusions.

**Table 4.** Count of data cleaning stage design factors, decision alternatives selected by each AI-DPA, and identification of which AI-DPA systems provided explicit justifications for their selection.

**Stage 3: Data cleaning** *Design factors* and decision alternatives for each	DI^19^	Duke Model 1^20^	DEWS^21^	APPROVE^22^	eCART^4^	MEWS++^5^	AAM^16^	Duke Model 2^23^	CHARTwatch^24^	MC-EWS^25^	CONCERN^26^^,^^27^	eCARTv5^28^	Total
*[3.1] Data error handling*													
Specified vital sign ranges			1				1	1		1		1	5
Trim values < 1% and > 99%									1				1
Not reported or not done	1	1		1	1	1					1		6
**Decision justifications** ^a^												**1**	**1**
Prior work												1	1
*[3.2] Missing value imputation*													
Median value (dataset)			1		1	1							3
Mean value (dataset)									1				1
Median matched for age, sex	1												1
Normal value (specified)								1					1
Random forest patient match				1									1
Left blank		1								1		1	3
Not reported							1				1		2
**Decision justifications**		**1**		**1**	**1**	**1**							**4**
Prior work				1	1	1							3
To suit algorithm		1											1
*[3.3] Training data exclusions*													
Normal (child, non-ward)	1	1	1	1	1	1	1			1		1	9
Palliative/hospice admissions	1					1					1		3
Psychiatry admissions						1				1			2
Maternity admissions						1	1						2
Rehabilitation admissions										1			1
Admissions for research										1			1
Non-general medical wards								1	1				2
Stays removed < 8, 24 h, or > 40 h									1		1		2
Outcome occurred before bed allocation		1		1									2
Outcome occurred before 1st AI-DPA assessment (4 h after admission)				1									1
Outcome within 30 min of admission			1										1
Stays censured after 7, 15, 60 days							1	1			1		3
**Decision justifications^a^**						**1**	**1**	**1**	**1**				**4**
Low frequency of escalation or lack of monitoring in the unit						1							1
To reduce training bias							1		1				2
Clinical reason: lower monitoring								1					1

a Bold 1 indicates the system has a decision justification and the specific justification is itemised below.

### AI-DPA development stage 4: data labeling

We found 10 distinct training label outcomes, of which AI-DPAs used a median of 2.5 binary outcomes (range 1-5) as a single composite label for deterioration (see Table 5). Nearly all (92%, n = 11) AI-DPAs used in-hospital death, 75% (n = 9) used unplanned transfer to the ICU, of which MC-EWS further qualified the timing of the ICU stay,^25^ and 33% (n = 4) used cardiac arrest. The other 6 outcomes were used by 3 or less AI-DPAs. Half the AI-DPIs provided justifications for outcome selection with 4 drawing on prior work,^16^^,^^23^^,^^25^^,^^28^ and then one each selecting outcomes which enabled earlier prediction,^22^ were most meaningful in terms of patients left on the ward too long,^4^ or captured most forms of outcomes.^23^

**Table 5.** Count of data labeling stage design factors, decision alternatives selected by each AI-DPA, and identification of which AI-DPA systems provided explicit justifications for their selection.

**Stage 4: Data labeling** *Design factors* and decision alternatives for each	DI^19^	Duke Model 1^20^	DEWS^21^	APPROVE^22^	eCART^4^	MEWS++^5^	AAM^16^	Duke Model 2^23^	CHARTwatch^24^	MC-EWS^25^	CONCERN^26^^,^^27^	eCARTv5^28^	Total
*[4.1] Outcome label selection*													
In hospital death	1	1	1	1	1	1	1	1	1		1	1	11
Unplanned transfer to ICU (UPICU)	1				1	1		1	1	1	1	1	8
UPICU > 6 h stay or <6 h + death/theatre							1						1
Unplanned move to step-down unit						1			1				2
Unplanned return to operating theatre	1												1
Cardiac arrest			1		1					1	1		4
MET/RRT call	1									1	1		3
Intubation + mechanical ventilation > 48 h				1									1
Sepsis											1		1
Transfer to palliative care									1				1
	4	1	2	2	3	3	2	2	3	3	5	2	
**Decision justifications** ^a^				**1**	**1**		**1**	**1**		**1**		**1**	**6**
Prior work							1	1		1		1	4
Enable earlier prediction				1									1
Most meaningful outcome					1								1
Captures most forms of outcomes								1					1
*[4.2] Positive sample horizon to event*													
Within 6 h of outcome event						1							1
Within 12 h of outcome event							1	1					2
Within 24 h of outcome event										1	1	1	3
Within 5-24 h of outcome event			1		1								2
Within 48 h of outcome event				1					1				2
Not reported	1	1											2
**Decision justifications^a^**					**1**	**1**	**1**	**1**					**4**
Prior work					1								1
Clinical considerations: believable and actionable						1	1	1					3

MET: medical emergency team; RRT: rapid response team.

a Bold 1 indicates the system has a decision justification and the specific justification is itemised below.

In assigning training samples to a positive or negative outcome label for training purposes, where positive indicates deterioration, positive samples were assigned as those occurring 6,^5^,12,^16^^,^^23^,24,^25^^,^^26^^,^^28^,48,^22^^,^^24^ and between 0.5 and 24 hours^4^^,^^21^ before the first outcome event. Of the 10 AI-DPA reporting a positive sample horizon, 4 provided justifications for their selection, 3 for clinical reasons to provide actionable alerting periods or to align with staff shift timings,^5^^,^^16^^,^^23^ and 1 drew upon prior work.^4^

### AI-DPA development stage 5: feature engineering

**Figure 3.**
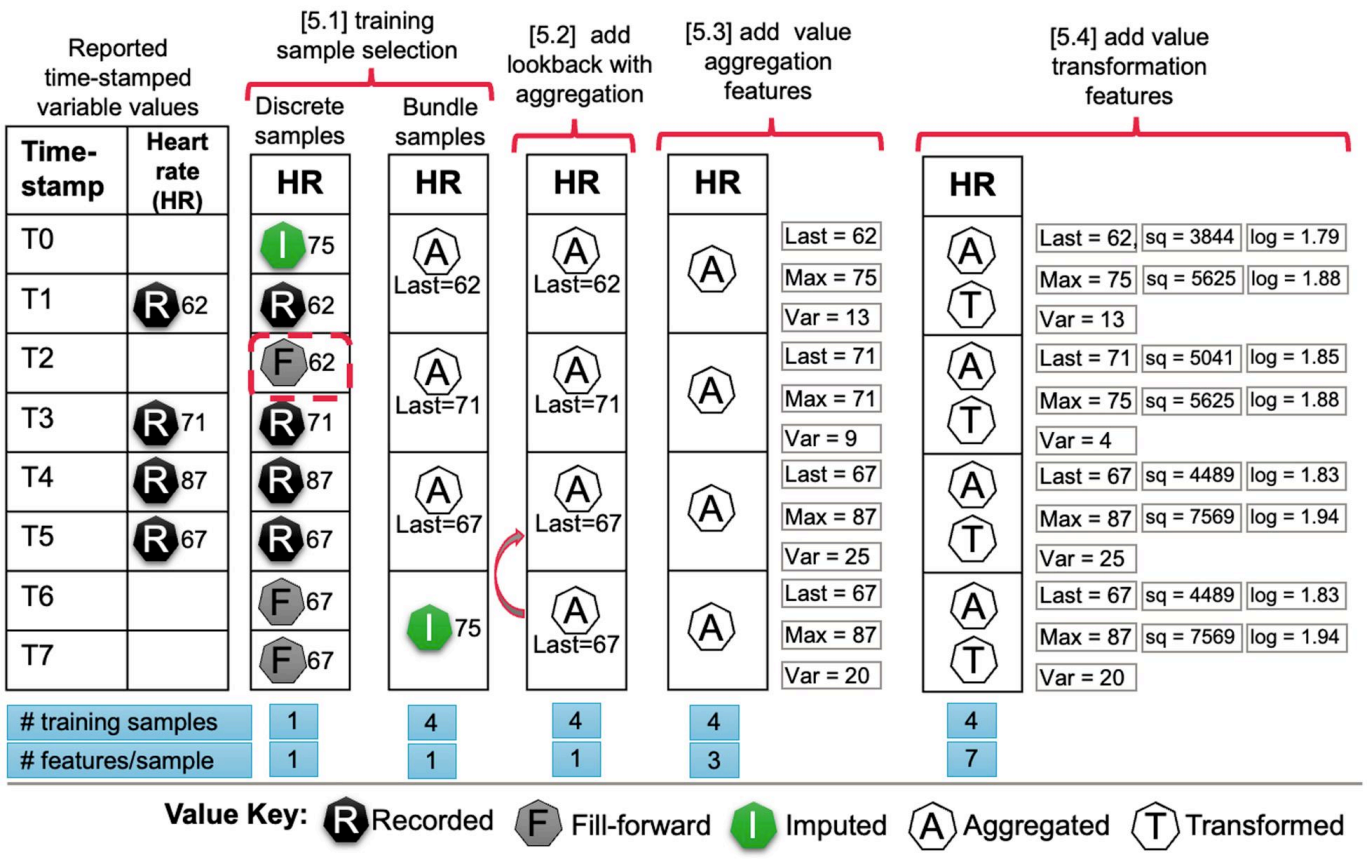
Explanatory example of how raw recorded values for a single variable, heart rate (HR), can be transformed into algorithm training sample features. Actual time-stamped recorded HR raw values are listed in the left-hand 2 columns. The remaining columns demonstrate example decision alternatives for each design factor, 5.1-5.4 labeled, at the top of the figure: [Factor 5.1] There are 2 alternatives. The first column demonstrates a “discrete” training sample selection where a single value at a given timestamp (in this case T2) is used for training. When a timestamp entry for HR is missing, the last prior value is brought forward (called “fill-forward,” denoted “F”) and if there is no prior value, then a value is imputed (denoted I). The second column under [5.1] demonstrates bundled (n = 2 time units) training samples with a “within-bundle” data lookback context applied and “last” recorded value aggregation, or imputed value if bundle value is missing; [Factor 5.2] Same as [5.1] bundle example, but adds a data lookback context of n = 3 time units when a bundle field is empty; [Factor 5.3] Same as [5.2], but adds maximum and variance (maximum—minimum) aggregated values over the lookback period as 2 new features per sample; and [Factor 5.4] Same as [5.3], but adds value transformations (squared and logarithmic) applied to the “last” value and “maximum” value aggregations, as 4 new features per sample.

AI-DPAs are typically trained by inputting to the algorithm a single sample, comprising a fixed size set of numeric features, derived from the selected variables, plus one binary outcome label (0 or 1) associated with that sample, for example death or survival This is repeated until the AI-DPA “learns” to classify samples into the correct binary outcome with acceptable accuracy. We found 4 design factors involved with transforming the raw data variables from the prepared dataset (step 3) to the final training dataset containing input samples. Figure 3 depicts how design factors can be applied to raw data values for a single exemplar variable, heart rate (HR), and what their impact on feature counts are for each sample.

In deciding on the training sample definition, half (n = 6) of AI-DPA^4^^,^^5^^,^^21^^,^^22^^,^^25^^,^^28^ used 5 different discrete sampling methods, 42% (n = 5) used bundle samples^16^^,^^19^^,^^20^^,^^23^^,^^24^ (see Table 6) with bundle time intervals ranging from 1 to 6 hours or through to admission time, while CONCERN did not report their method.^26^ Four of the 6 AI-DPA that reported decision justifications identified possible operational or algorithm development benefits^16^^,^^23^^,^^25^^,^^28^; 3 reported clinically related reasons, such as capturing trends or matching to vital sign frequency^5^^,^^16^^,^^19^; and 1 experimented in selecting a time interval for discrete sample selection.^4^

**Table 6.** Count of training data transformation design factors, decision alternatives selected by each AI-DPA, and identification of which AI-DPA studies provided explicit justifications for their selection.

**Stage 5: Feature engineering** *Design factors* and decision alternatives	DI^19^	Duke Model 1^20^	DEWS^21^	APPROVE^22^	eCART^4^	MEWS++^5^	AAM^16^	Duke Model 2^23^	CHARTwatch^24^	MC-EWS^25^	CONCERN^26^^,^^27^	eCARTv5^28^	Total
*[5.1] Training sample definition*													
1 sample per 1-h interval (bundle)							1						1
1 sample per 2-h interval (bundle)								1					1
1 sample per 6-h interval (bundle)									1				1
1 sample per varied-hour interval (bundle)	1												1
1 sample across all ED stay data (bundle)		1											1
1 discrete sample per 4-h interval						1							1
1 discrete sample per 8-h interval					1							1	2
1 discrete sample from each admission										1			1
N random discrete samples from each admission				1						1			2
All discrete raw samples			1										1
Unclear/not reported											1		1
**Decision justifications** ^a^	**1**				**1**	**1**	**1**	**1**		**1**		**1**	**7**
Operational or development benefit							1	1		1		1	4
Clinical reasons: trends, vital frequency	1					1	1						3
Experimentation					1								1
*[5.2] Data look-back context*													
Past 8 h			1										1
Past 24/48 h										1	1	1	3
Past to admission				1						1		1	3
Past 12 months (inter-admission)							1						1
Bundle interval only						1							1
Bundle interval, else lookback 24 h						1	1						2
Bundle interval, else lookback 72 h							1						1
Bundle interval, else look-back to admission	1				1			1	1			1	5
Impute when no lookback data found	1		1	1	1	1		1	1				7
**Decision justifications^a^**								**1**				1	**2**
Clinical relevance of variables								1				1	2
*[5.3] Aggregations in data lookback context*													
First value recorded					1							1	2
Last value recorded	1						1	1				1	4
First in admission, Variance (last - 2nd last/3rd last/first in admission)	1											1	2
Minimum, maximum, and variance (Max.-Min.) in lookback interval		1					1			1		1	4
Mean/count/SD of values recorded		1										1	2
Not reported						1			1		1		3
**Decision justifications^a^**	**1**					**1**	**1**						**3**
Pattern/trend capture	1					1	1						3
*[5.4] Final value transformation*													
No further modification	1	1	1	1	1	1	1	1	1	1		1	11
Squared					1		1	1					3
Cubed, logarithmic, log(value/(1-value))							1						1
Value present flag							1		1				2
Convert to 1-hot categories					1	1	1						3
Convert values to 1-hot categories: (normal, high, low, not ordered)								1					1
Splined into multiple features					1			1					2
Unclear/not reported											1		1
**Decision justifications**						**1**		**1**	**1**				**3**
To capture non-linearity of variables						1							1
To handle missing variable values									1				1
Experiments to optimize effectiveness								1					1

a Bold 1 indicates the system has a decision justification and the specific justification is itemised below.

Brajer et al’s AI-DPA^20^ was the only system that presented a single, hospital-level prediction at the end of the emergency department (ED) stay, whereas all other systems used time-horizons to make continuous predictions during the hospital stay. The time-horizon systems used one or more of 8 lookback contexts (design factor 5.2) that define how far back one looks to find a variable value for aggregation, eg, for a minimum variable value, or to bring a variable forward when bundle or discrete time-point variable data is missing. Five AI-DPAs^4^^,^^19^^,^^23^^,^^24^^,^^28^ looked within the bundle first and reached as far back as the admission time to find missing values, while all other lookback contexts were used by 3 or fewer AI-DPAs. Only 2 AI-DPA reported a justification which was to account for variation in clinical relevance of the variable within a timeframe.^23^^,^^28^

Seven AI-DPA reported variable aggregations within the data lookback context (design factor 5.3), noting a single variable was, in many cases, aggregated in multiple ways (see Figure 3), resulting in additional training features per variable. The most commonly used aggregations were “last” value recorded^16^^,^^19^^,^^23^^,^^28^ and “minimum,” “maximum,” and their difference (“variance”).^16^^,^^20^^,^^25^^,^^28^ Of the 7 AI-DPA using aggregations, 3 justified their selections, which was to capture patterns or trends in the longitudinal time-based data.^5^^,^^16^^,^^19^

Five (43%) of the AI-DPAs^4^^,^^5^^,^^16^^,^^23^^,^^24^ further transformed the aggregated or discrete variable values (design factor 5.4) and multiple transformations were also applied to the same variable resulting in one additional feature per transformation (see Figure 3**)** as used in the AAM algorithm which combined raw variable values with squares, cubes, and logarithmic transformations.^16^ Squared variables were used by 3 AI-DPAs^4^^,^^16^^,^^23^ and 4 used 1-hot categorical variable transformations—converting an n-categorical variable to n-binary (0,1) feature values.^4^^,^^5^^,^^16^^,^^23^ Of the 5 AI-DPAS using value transformation, 3 provided justifications, including eCART, to capture non-linearity,^4^ another AI-DPI to handle missing variable values^23^ and AAM on the basis of experimentation.^16^

### AI-DPA development stage 6: model training

Referring to Table 7, parameter or hyper-parameter optimization (design factor 6.1) was not reported by half (n = 6) the AI-DPA,^5^^,^^22^^,^^24–26^^,^^28^ 3 used logistic regression algorithms requiring no optimization, leaving 3 reporting optimization methods.^4^^,^^20^^,^^21^ Only eCART referenced prior work for their method.^4^ Half (n = 6) the AI-DPA did not report their training sample selection techniques (Design factor 6.2) and of those that did, 4 (33%) used different under-sampling^5^^,^^16^^,^^25^^,^^28^ techniques and 1 each used over-sampling^21^ and algorithm parameter weighting.^4^ AAM^16^ and eCARTv5^28^ selected methods based on prior work and eCART^4^ on prior experimentation.

**Table 7.** Count of model training stage design factors, decision alternatives selected by each deployed AI-DPA, and identification of which AI-DPA systems provided explicit justifications for their selection.

**Stage 6: Model training** *Design factor* and decision alternatives	DI^24^	Duke Model 1^25^	DEWS^26^	APPROVE^27^	eCART^4^	MEWS++^5^	AAM^16^	Duke Model 2^28^	CHARTwatch^29^	MC-EWS^30^	CONCERN^31^^,^^32^	eCARTv5^33^	Total
*6.1 Parameter optimization*													
Parameter selection identified		1	1		1								3
Not reported (excluding log. regression)				1		1			1	1	1	1	6
**Decision justifications** ^a^					**1**								**1**
Prior work					1								1
*6.2 Class sampling technique*													
Over-sampling (positive outcome)			1										1
Under-sampling (no outcome)						1	1			1		1	4
Weighting parameters					1								1
Not reported	1	1		1				1	1		1		6
**Decision justifications^a^**					**1**		**1**					**1**	**3**
Prior work							1					1	2
Experimentation					1								1

a Bold 1 indicates the system has a decision justification and the specific justification is itemised below.

## Discussion

We sought to break down AI-DPA development into constituent design factors that could impact AI-DPA accuracy, identify decision alternatives in relation to those design factors, and ascertain the explicit justifications given by development teams for each decision. We hypothesized this would yield useful summary information about the development of deployed AI-DPA, which is relevant to both future AI-DPA developers and healthcare organizations seeking to select and implement them.

The first key finding was that 13 design factors (see Figure 2) and 315 associated decision alternatives were identified, with AI-DPAs reporting 128 of 156 (82%) possible design factor decisions (N = 156 possible decisions = 12 AI-DPA × 13 design factors). Of those 128 reported decisions, justifications were provided for 44%. AI-DPA decisions varied widely across all factors with little or no evidence supporting most decisions. The second key finding was development and evaluation of most AI-DPAs occurring with minimal application of, or comparison with, prior work. This latter finding underscores the limited evidence base supporting development of deployed AI-DPAs and needs addressing as a priority for future work.

### Similarities

Where at least half (n = 6) of the AI-DPAs made the same design factor decision, logistic regression algorithms were selected (n = 6), 12% of all identified variables (6 vital signs, 17 laboratory results, 3 non-clinical variables) were used, data imputation was used (n = 7) to handle missing values, the same defined set of data exclusions were applied (n = 9), raw or aggregated variable values were used as features without further transformation (n = 11) for at least one of each DPA-AI’s variables, and training outcomes included in-hospital death (n = 11) and unplanned ICU transfer (n = 8).

### Differences

Where design factor alternatives were chosen by not more than 3 of 12 AI-DPA, the noted differences related to 86% (N = 7) of algorithm alternatives, 84% (N = 226) of variable alternatives, 100% (N = 6) of data imputation alternatives, 94% (N = 16) of training data exclusion alternatives, 70% (N = 10) of outcome label alternatives, 100% (N = 5) of training label horizon alternatives, 100% (N = 10) of training sample definition alternatives, 89% (N = 9) of data lookback alternatives, 92% (N = 12) of aggregation alternatives, 89% (N = 9) of final variable value transformation alternatives, and 67% (N = 3) of training sampling technique alternatives.

These findings prompt several questions. First, why relevant prior research was not used in 90% of reported design decisions? Prior research was only applied to 4 AI-DPA for outcome label selection^16^^,^^23^^,^^25^^,^^28^; to 3 AI-DPA for data imputation method selection^4^^,^^5^^,^^22^; to 2 AI-DPA for selecting under-sampling techniques^16^^,^^28^; to eCART for variable selection, positive label horizon, and parameter optimization methods^28^; and to eCARTv5 for data error handling methods.^28^ Rather than reference relevant prior research, some AI-DPA developers used experimentation, which accounted for 10% (N = 128) of reported decisions and used specifically for algorithm selection, variable selection, training sample definition, value transformations, and training sampling technique.

Second, why is there no state-of-the-art (SOTA) standard for AI-DPAs in guiding design factor decisions? All 12 included AI-DPA were built from scratch, with no reported reference to a SOTA standard, and only the AAM AI-DPA compared their accuracy with a prior AI-DPA (eCART).^16^ Ten AI-DPAs compared their systems to rule-based baselines, namely NEWS^34^ and MEWS,^35^ while 2 others performed no comparisons.^20^^,^^24^ Without a SOTA standard, decision makers cannot evaluate the relative value of a current or potential AI-DPA.

Third, why are AI-DPAs developed so differently? We hypothesize AI-DPA development decisions are influenced by several dependencies: (i) Algorithm selection is limited in order to avoid complexity,^16^^,^^20^^,^^23^ for example, computational limitations of EMR systems enforcing use of logistic regression^23^; (ii) Available variables are limited in number or different or AI-DPA developers prefer widely used EMR variables^4^^,^^20–23^; (iii) Varying levels of missingness of variables changes the extent of data imputation, which possibly impacts their predictive value within the training samples; (iv) Varying frequency of variable capture, eg, vital sign versus lab variables, impacts bundle interval size^5^; (v) Different presenting conditions and comorbidities of admitted patients impact reasons for deterioration, which influences the relative importance of different variables; and (vi) Outcomes such as cardiac arrest or ventilation data are not digitally captured, thereby restricting AI-DPA training.

Finally, why do agreed training outcome labels for deterioration remain elusive? The commonly used training labels—death, ICU transfer, and cardiac arrest—are fraught with known problems: they represent late-stage deterioration, reducing the time available for clinicians to take effective preventive actions; algorithm accuracy varies depending on the specific outcome chosen; ICU transfer policies vary between hospitals; death outcome labels include “expected” patient deaths, including palliated patients; and cardiac arrests are very rare.^6^^,^^36–38^ Moreover, patients who deteriorate on the ward but then recover are incorrectly labeled as non-deteriorated, meaning models are both miss-trained and miss-evaluated on this cohort. If the training outcome label is a poor proxy for actual patient deterioration, then predictions made by the AI-DPA trained on it may have limited clinical impact, as suggested by current evidence.^39^ Universally accepted outcome labels are urgently required.

### Potential areas of research to improve AI-DPA accuracy

We conclude that AI-DPA developers made design factor decisions in response to limitations imposed by their local environment, particularly dataset capabilities (as defined above). This context-specific constraint means it is impossible for external parties to evaluate, for their own context, the benefit of one decision over another for a given design factor or determine the relative importance of different design factors. Managers within different hospitals need guiding principles for handling the real-world challenges (itemized above) each has to contend with when implementing one or other AI-DPA. Specifically, the following key operational questions remain outstanding, which require further research:

1. *Which algorithms are most accurate*? While considerable prior work compares results from existing automated prediction algorithms, mostly non-AI,^3^^,^^12^^,^^40^^,^^41^ they do not control for differences in design factors, nor use a common dataset. Only Edelson et al^42^ compared performance, using the same dataset, between the Epic Deterioration Index (not reported here for lack of development details) and eCARTv5.^28^ Other algorithm benchmarking studies use high frequency ICU data with variable selection for multiple prediction tasks, including deterioration defined as mortality.^43^^,^^44^
2. *Which variables impact most on accuracy and can a common benchmark variable set be devised that most EMR systems can provide*? Gerry et al^45^ reported the most common variables used by AI-DPAs, both implemented and in silico, but did not make accuracy comparisons. Muralitharan et al^12^ also reported variable usage and provided an overview accuracy comparison, but DPAs were evaluated on different datasets, thus negating fair comparisons, and no other design factors were controlled for. Other studies have focused on demonstrating superior model performance by adding some specific variables.^46^^,^^47^
3. *What is the impact of different data cleaning methods and which are optimal*? Budach et al^48^ comprehensively evaluated the impact of data quality issues, such as missingness, on algorithm performance, but this was not specific to healthcare and did not consider domain-specific choices around data error handling methods, dataset exclusions, or imputation methods. Other studies have considered imputation methods but not their impact on algorithms.^49^^,^^50^
4. *What deterioration outcome is best for a training and evaluation label*? Considerable debate continues with no standard currently in existence.^37^^,^^38^
5. *What is the impact of changing the positive label time horizon and what is optimal for algorithm training*? Unlike Zargoush et al^51^ who investigated the impact on sepsis prediction algorithms of the size of the prediction window and data observation window (ie, data recency), no similar study exists for deterioration prediction.
6. *What is the impact of bundling versus discrete training data sampling and how does this vary with the bundle interval*?
7. *What is the impact of data recency and the benefit of applying more complex data lookback regimes together with different data aggregation methods*? Refer to comment on item (4) above.
8. *Does transforming selected variables improve accuracy and how might this change the benchmark variable dataset*?
9. *How do the answers to (1) to (7) change when typical dataset limitations change, for example, with different levels of data missingness or data capture frequency, or differences in patient mix or ability to capture variables and outcomes*?

Answering each of these question requires research, but in order to do it baselines need to be established and ablation studies conducted to assess the relative merits of different decisions relating to each design factor, and the relative impact of differences between design factors. For example, whether adding serum potassium level as a variable is helpful or not and under what circumstances, or whether variable changes are more or less important than changing the lookback contexts.

### Strengths and limitations

The strength of this review is the in-depth extraction and analysis of multiple design factors relating to AI-DPA that have actually been deployed in real-world clinical practice. Most of our findings are novel and have been brought together as one integrated report, although a few findings overlap with those of prior reviews but which studied non-deployed AI-DPAs.^12^

Study limitations were that we relied on analysis of only12 implemented AI-DPAs that have published development studies, and it is possible many other deployed AI-DPAs, either unpublished or not found by our search methods were missed. However, this study set out to consider similarities and differences in design decisions, and the 12 AI-DPAa deployed at scale provided extensive data to draw upon.

## Conclusions

The study of the development reports of 12 deployed AI-DPA shows considerable variation between the choices made for design factors, all potentially impacting algorithm accuracy. This inconsistency in design decisions and their rationales, the lack of reference in most cases to past work, the absence of a benchmark dataset of variables or SOTA algorithms as comparative reference standards, and non-standardized outcome labels for algorithm training negate any attempt to compare the accuracy of different AI-DPA and prevent hospitals from making an informed choice as to which algorithm they should select for implementation. These deficits need to be addressed as a priority in future research.

## Supplementary Material

ocae321_Supplementary_Data

## Data Availability

There is no data used in this study.
