# Supplementary material for: Factors underpinning the performance of implemented artificial intelligence-based patient deterioration prediction systems: reasons for selection and implications for hospitals and researchers
Source: J Am Med Inform Assoc. 2025 Jan 16;32(3):492–509. doi: 10.1093/jamia/ocae321 (PMC11833469; doi:10.1093/jamia/ocae321)
Supplement: ocae321_Supplementary_Data [file ocae321_supplementary_data.docx]

Supplementary appendices.

## Appendix A: Complete variable use tables

### Table A.1: Vital sign and nurse-captured variables

| Variable | Bell 2021 (DI) | Brajer 2020 | Kwon et al (DEWS) | Dziadzko 2018 (APPROVE) | Churpek 2014 (eCART) | Kia 2020 (MEWS++) | Kipnis 2016 (AAM) | O'Brien 2019 | Pou-Prom 2022 (CHARTwatch) | Romero-Brufau 2021 (MC-EWS) | Rossetti, 2024 (CONCERN) | Churpek, 2024 (eCARTv5) | Total count |
| --- | --- | --- | --- | --- | --- | --- | --- | --- | --- | --- | --- | --- | --- |
| Systolic blood pressure | U | U | U | U | U | U | U | U | U | U |  | U | 11 |
| Respiratory rate | U | U | U | U | U | U | U | U | U | U |  | U | 11 |
| Body temperature | U | U | U | U | U | U | U | U | U | U |  | U | 11 |
| Heart rate/pulse rate | U | U | U | U | U | U | U | U | U | U |  | U | 11 |
| Diastolic blood pressure | U | U |  | U | U | U | U | U | U | U |  | U | 10 |
| Oxygen saturation | U | U |  | U | U | U | U | U | U | U |  | U | 10 |
| State of conciousness (AVPU) | U |  |  |  | U | U |  | U |  |  |  | U | 5 |
| Supplemental oxygen |  | U |  | U |  |  |  | U |  | U |  |  | 4 |
| Pulse rate (specified in addition to HR) |  | U |  |  |  |  |  |  |  | U |  |  | 2 |
| Fraction of inspired oxygen |  |  |  |  |  |  |  |  | U |  |  | U | 2 |
| Neurological/mental status |  | U |  |  |  |  | U |  |  |  |  |  | 2 |
| Richmond agitation & sedation score |  |  |  | U |  |  |  |  |  | U |  |  | 2 |
| Braden activity |  |  |  |  |  |  |  |  |  | U |  | U | 2 |
| Braden mobility |  |  |  |  |  |  |  |  |  | U |  | U | 2 |
| Braden nutrition |  |  |  |  |  |  |  |  |  | U |  | U | 2 |
| Braden sensory perception |  |  |  |  |  |  |  |  |  | U |  | U | 2 |
| Respiratory pattern (not defined) |  |  |  |  |  |  |  |  |  | U |  |  | 1 |
| Breaths per minute |  |  |  |  |  |  |  |  |  | U |  |  | 1 |
| Mean arterial pressure |  |  |  |  |  | U |  |  |  |  |  |  | 1 |
| Oxygen therapy: no definition |  |  |  |  |  |  |  |  |  | U |  |  | 1 |
| Cardiac regularity (string) - not def. |  |  |  |  |  | U |  |  |  |  |  |  | 1 |
| Respiratory pattern (string) - not def. |  |  |  |  |  | U |  |  |  |  |  |  | 1 |
| Tenderness (string) - not def. |  |  |  |  |  | U |  |  |  |  |  |  | 1 |
| Level of activity (string) - not def. |  |  |  |  |  | U |  |  |  |  |  |  | 1 |
| Pain intensity with movement |  |  |  |  |  |  |  |  | U |  |  |  | 1 |
| Pain intensity at rest |  |  |  |  |  |  |  |  | U |  |  |  | 1 |
| Hendrichs fall score |  |  |  |  |  |  |  |  |  | U |  |  | 1 |
| Braden skin score |  |  |  |  |  |  |  |  |  | U |  |  | 1 |
| Braden friction and shear |  |  |  |  |  |  |  |  |  |  |  | U | 1 |
| Braden moisture |  |  |  |  |  |  |  |  |  |  |  | U | 1 |
| Braden total score |  |  |  |  |  |  |  |  |  |  |  | U | 1 |
| Cllinical institute withdrawal assessment of alcohol |  |  |  |  |  |  |  |  | U |  |  |  | 1 |
| Braden falls risk score |  |  |  |  |  |  |  |  | U |  |  |  | 1 |
| times incontinent |  |  |  |  |  |  |  |  | U |  |  |  | 1 |
| Disorientation (yes/no) |  |  |  |  |  |  |  |  |  |  |  | U | 1 |
| Sum of urine output over the last 24 hours |  |  |  |  |  |  |  |  |  |  |  | U | 1 |
| Nursing note content: abdominal pain |  |  |  |  |  |  |  |  |  |  | U |  | 1 |
| Nursing note content: abnormal heart rhythm |  |  |  |  |  |  |  |  |  |  | U |  | 1 |
| Nursing note content: abnormal mental state |  |  |  |  |  |  |  |  |  |  | U |  | 1 |
| Nursing note content: abnormal rate, rhythm, depth and effort of respirations |  |  |  |  |  |  |  |  |  |  | U |  | 1 |
| Nursing note content: abnormal temperature |  |  |  |  |  |  |  |  |  |  | U |  | 1 |
| Nursing note content: back pain |  |  |  |  |  |  |  |  |  |  | U |  | 1 |
| Nursing note content: chest pain |  |  |  |  |  |  |  |  |  |  | U |  | 1 |
| Nursing note content: communication problem |  |  |  |  |  |  |  |  |  |  | U |  | 1 |
| Nursing note content: diagnosis related with infection |  |  |  |  |  |  |  |  |  |  | U |  | 1 |
| Nursing note content: deficit of circulation |  |  |  |  |  |  |  |  |  |  | U |  | 1 |
| Nursing note content: fall risk |  |  |  |  |  |  |  |  |  |  | U |  | 1 |
| Nursing note content: fluid volume alteration |  |  |  |  |  |  |  |  |  |  | U |  | 1 |
| Nursing note content: general concern |  |  |  |  |  |  |  |  |  |  | U |  | 1 |
| Nursing note content: headache |  |  |  |  |  |  |  |  |  |  | U |  | 1 |
| Nursing note content: improper renal function |  |  |  |  |  |  |  |  |  |  | U |  | 1 |
| Nursing note content: medication related infection |  |  |  |  |  |  |  |  |  |  | U |  | 1 |
| Nursing note content: monitoring |  |  |  |  |  |  |  |  |  |  | U |  | 1 |
| Nursing note content: mood disorder |  |  |  |  |  |  |  |  |  |  | U |  | 1 |
| Nursing note content: musculoskeletal pain |  |  |  |  |  |  |  |  |  |  | U |  | 1 |
| Nursing note content: pain level |  |  |  |  |  |  |  |  |  |  | U |  | 1 |
| Nursing note content: violence gesture |  |  |  |  |  |  |  |  |  |  | U |  | 1 |
| Vital sign frequency: heart rate measurement |  |  |  |  |  |  |  |  |  |  | U |  | 1 |
| Vital sign frequency: respiratory rate measurement |  |  |  |  |  |  |  |  |  |  | U |  | 1 |
| Vital sign frequency: blood pressure measurement |  |  |  |  |  |  |  |  |  |  | U |  | 1 |
| Vital sign frequency: temperature measurement |  |  |  |  |  |  |  |  |  |  | U |  | 1 |
| Vital sign frequency: SpO2 measurement |  |  |  |  |  |  |  |  |  |  | U |  | 1 |
| Vital sign frequency: All 5 vital measurements taken at same time |  |  |  |  |  |  |  |  |  |  | U |  | 1 |
| Vital sign frequency: only 1 vital measurement taken |  |  |  |  |  |  |  |  |  |  | U |  | 1 |
| Nursing note frequency |  |  |  |  |  |  |  |  |  |  | U |  | 1 |
| Vital sign comment frequency: heart rate |  |  |  |  |  |  |  |  |  |  | U |  | 1 |
| Vital sign comment frequency: respiratory rate |  |  |  |  |  |  |  |  |  |  | U |  | 1 |
| Vital sign comment frequency: blood pressure |  |  |  |  |  |  |  |  |  |  | U |  | 1 |
| Vital sign comment frequency: temperature |  |  |  |  |  |  |  |  |  |  | U |  | 1 |
| Vital sign comment frequency: SpO2 |  |  |  |  |  |  |  |  |  |  | U |  | 1 |
| PRN medication administered |  |  |  |  |  |  |  |  |  |  | U |  | 1 |
| Scheduled medication withheld |  |  |  |  |  |  |  |  |  |  | U |  | 1 |

### Table A.2: Laboratory variables

| Variable | Bell 2021 (DI) | Brajer 2020 | Kwon et al (DEWS) | Dziadzko 2018 (APPROVE) | Churpek 2014 (eCART) | Kia 2020 (MEWS++) | Kipnis 2016 (AAM) | O'Brien 2019 | Pou-Prom 2022 (CHARTwatch) | Romero-Brufau 2021 (MC-EWS) | Rossetti, 2024 (CONCERN) | Churpek, 2024 (eCARTv5) | Total count |
| --- | --- | --- | --- | --- | --- | --- | --- | --- | --- | --- | --- | --- | --- |
| Blood urea nitrogen/Urea | U | U |  | U | U | U | U | U | U | U |  | U | 10 |
| White blood cell count | U | U |  | U | U | U | U | U | U | U |  | U | 10 |
| Sodium |  | U |  | U | U | U | U | U | U | U |  | U | 9 |
| Creatinine |  | U |  | U | U | U | U |  | U | U |  | U | 8 |
| Total bilirubin |  | U |  | U | U | U |  | U | U | U |  | U | 8 |
| Potassium |  | U |  | U | U | U |  | U | U | U |  | U | 8 |
| Anion gap (serum) |  |  |  | U | U |  | U | U | U | U |  | U | 7 |
| Bicarbonate |  |  |  | U | U | U | U |  | U | U |  | U | 7 |
| Glucose |  | U |  | U | U |  | U |  | U | U |  | U | 7 |
| Lactate (arterial blood gas) |  | U |  | U |  | U | U | U | U | U |  |  | 7 |
| Albumin (blood test - serum) |  | U |  | U | U |  |  | U | U | U |  | U | 7 |
| Haemoglobin | U |  |  | U | U | U |  |  | U | U |  | U | 7 |
| Platelet |  | U |  |  | U | U |  | U | U | U |  | U | 7 |
| Haematocrit/Haematocrit |  | U |  | U |  | U | U | U | U |  |  |  | 6 |
| Partial pressure oxygen (pO2) |  | U |  | U |  |  |  | U | U | U |  | U | 6 |
| Aspartate aminotransferase |  | U |  |  | U |  |  | U | U | U |  | U | 6 |
| International normalised ratio |  | U |  |  |  | U |  | U | U | U |  | U | 6 |
| Troponin |  | U |  |  |  |  | U | U | U | U |  |  | 5 |
| Partial pressure carbon dioxide (CO2) - arterial specified |  | U |  | U |  |  |  | U | U |  |  | U | 5 |
| Magnesium |  | U |  |  |  |  |  | U | U | U |  | U | 5 |
| pH (acidity) test – arterial |  | U |  | U |  |  |  | U | U |  |  | U | 5 |
| Calcium |  |  |  | U | U |  |  |  | U | U |  | U | 5 |
| Lactate (Venous blood gas) |  |  |  | U |  | U |  | U | U | U |  |  | 5 |
| Partial pressure carbon dioxide (CO2) - venous specified |  | U |  |  |  |  |  | U | U |  |  | U | 4 |
| Alanine aminotransferase |  | U |  |  |  |  |  | U | U | U |  |  | 4 |
| Alkaline phosphatase, |  |  |  |  | U |  |  |  | U | U |  | U | 4 |
| Neutrophils (also called bands) |  | U |  |  |  |  |  |  | U | U |  | U | 4 |
| C-reactive protein |  | U |  |  |  |  |  | U | U | U |  |  | 4 |
| Erythrocyte sedimentation rate |  | U |  |  |  |  |  | U | U | U |  |  | 4 |
| pH (acidity) test - venous |  | U |  |  |  |  |  | U | U |  |  | U | 4 |
| Chloride |  |  |  | U |  | U |  |  | U |  |  | U | 4 |
| Estimated glomerular filtration rate | U |  |  |  |  | U |  |  |  | U |  |  | 3 |
| Ammonia |  | U |  |  |  |  |  | U |  | U |  |  | 3 |
| Creatine Kinease |  | U |  |  |  |  |  | U | U |  |  |  | 3 |
| Total protein (albumin + globulin) |  |  |  |  | U |  |  |  | U |  |  | U | 3 |
| Lactate dehydrogenase |  | U |  |  |  |  |  | U | U |  |  |  | 3 |
| Phosphorus |  |  |  |  |  |  |  |  | U | U |  | U | 3 |
| Activated partial thomboplastin time |  |  |  |  |  |  |  |  | U | U |  | U | 3 |
| Lipase |  |  |  |  |  |  |  |  | U | U |  | U | 3 |
| Bicarbonate arterial |  | U |  |  |  |  |  |  | U |  |  |  | 2 |
| Bicarbonate (venous) |  | U |  |  |  |  |  |  | U |  |  |  | 2 |
| Direct bilirubin |  |  |  |  |  |  |  |  | U | U |  |  | 2 |
| Red blood cell distribution width |  |  |  |  |  |  |  |  | U |  |  | U | 2 |
| Eosinophils |  |  |  |  |  |  |  |  | U |  |  | U | 2 |
| Bandemia test |  |  |  |  |  |  |  | U |  |  |  | U | 2 |
| Creatine Kinease MB |  | U |  |  |  |  |  | U |  |  |  |  | 2 |
| D-dimer |  | U |  |  |  |  |  | U |  |  |  |  | 2 |
| Fibrinogen |  | U |  |  |  |  |  | U |  |  |  |  | 2 |
| pH (acidity) test - unspecified |  |  |  |  |  |  |  |  | U | U |  |  | 2 |
| Base excess |  |  |  |  |  | U |  |  | U |  |  |  | 2 |
| Calcium ionized |  |  |  |  |  |  |  |  | U | U |  |  | 2 |
| Blood cultures ordered |  | U |  |  |  |  |  | U |  |  |  |  | 2 |
| Amylase |  |  |  |  |  |  |  |  | U | U |  |  | 2 |
| Lymphocytes |  |  |  |  |  |  |  |  | U |  |  | U | 2 |
| Monocytes |  |  |  |  |  |  |  |  | U |  |  | U | 2 |
| Mean corpuscular volume |  |  |  |  |  |  |  |  | U |  |  | U | 2 |
| Point-of-care glucose |  |  |  |  |  |  |  |  | U |  |  |  | 1 |
| Glucose arterial |  |  |  |  |  |  |  |  | U |  |  |  | 1 |
| Haematocrit arterial calculation |  |  |  |  |  |  |  |  | U |  |  |  | 1 |
| Sodium arterial |  |  |  |  |  |  |  |  | U |  |  |  | 1 |
| Red blood cells |  |  |  |  |  |  |  |  | U |  |  |  | 1 |
| Partial pressure carbon dioxide (CO2) - unspecified |  |  |  |  |  |  |  |  |  | U |  |  | 1 |
| Partial pressure oxygen (pO2) - venous |  |  |  |  |  |  |  |  | U |  |  |  | 1 |
| Glycosylated Haemoglobin (also hba1c) |  |  |  |  |  |  |  |  | U |  |  |  | 1 |
| Mean corpuscular haemoglobin |  |  |  |  |  |  |  |  | U |  |  |  | 1 |
| Mean corpuscular haemoglobin concentration |  |  |  |  |  |  |  |  | U |  |  |  | 1 |
| Basophils |  |  |  |  |  |  |  |  | U |  |  |  | 1 |
| H ion (arterial) |  |  |  |  |  |  |  |  | U |  |  |  | 1 |
| H ion (venous) |  |  |  |  |  |  |  |  | U |  |  |  | 1 |
| Mean platelet |  |  |  |  |  |  |  |  | U |  |  |  | 1 |
| Base excess - venous |  |  |  |  |  |  |  |  | U |  |  |  | 1 |
| Potassium arterial |  |  |  |  |  |  |  |  | U |  |  |  | 1 |
| Potassium random |  |  |  |  |  |  |  |  | U |  |  |  | 1 |
| Calcium ionized (corrected to ph7.4) |  |  |  |  |  |  |  |  | U |  |  |  | 1 |
| Calcium ionized arterial |  |  |  |  |  |  |  |  | U |  |  |  | 1 |
| Lactate unspecified |  |  |  |  |  |  |  |  |  |  |  | U | 1 |
| Chloride random |  |  |  |  |  |  |  |  | U |  |  |  | 1 |
| Total base |  |  |  |  |  |  |  |  |  | U |  |  | 1 |
| Prothrombin time |  |  |  |  |  |  |  |  | U |  |  |  | 1 |
| Acetaminophen level |  |  |  |  |  |  |  |  | U |  |  |  | 1 |
| Aortic root |  |  |  |  |  |  |  |  | U |  |  |  | 1 |
| Salicylate level |  |  |  |  |  |  |  |  | U |  |  |  | 1 |
| Vitamin B12 level |  |  |  |  |  |  |  |  | U |  |  |  | 1 |
| NT-proBNP |  |  |  |  |  |  |  |  | U |  |  |  | 1 |
| Ethanol |  |  |  |  |  |  |  |  | U |  |  |  | 1 |
| Iron total |  |  |  |  |  |  |  |  | U |  |  |  | 1 |
| Total iron binding capacity |  |  |  |  |  |  |  |  | U |  |  |  | 1 |
| Ferritin |  |  |  |  |  |  |  |  | U |  |  |  | 1 |
| Globulin |  |  |  |  |  |  |  |  | U |  |  |  | 1 |
| Immunoglobulin A |  |  |  |  |  |  |  |  | U |  |  |  | 1 |
| Left atrium |  |  |  |  |  |  |  |  | U |  |  |  | 1 |
| Measured arterial O2 saturation |  |  |  |  |  |  |  |  | U |  |  |  | 1 |
| Measured venous O2 saturation |  |  |  |  |  |  |  |  | U |  |  |  | 1 |
| Absolute metamyelocytes |  |  |  |  |  |  |  |  | U |  |  |  | 1 |
| Absolute myelocytes |  |  |  |  |  |  |  |  | U |  |  |  | 1 |
| Osmolality serum |  |  |  |  |  |  |  |  | U |  |  |  | 1 |
| Osmolality |  |  |  |  |  |  |  |  | U |  |  |  | 1 |
| Reticulocyte count |  |  |  |  |  |  |  |  | U |  |  |  | 1 |
| Specific gravity |  |  |  |  |  |  |  |  | U |  |  |  | 1 |
| Thyroid stimulating hormone |  |  |  |  |  |  |  |  | U |  |  |  | 1 |
| Urobilinogen |  |  |  |  |  |  |  |  | U |  |  |  | 1 |

### Table A.3: Non-clinical variables

| Variable | Bell 2021 (DI) | Brajer 2020 | Kwon et al (DEWS) | Dziadzko 2018 (APPROVE) | Churpek 2014 (eCART) | Kia 2020 (MEWS++) | Kipnis 2016 (AAM) | O'Brien 2019 | Pou-Prom 2022 (CHARTwatch) | Romero-Brufau 2021 (MC-EWS) | Rossetti, 2024 (CONCERN) | Churpek, 2024 (eCARTv5) | Total count |
| --- | --- | --- | --- | --- | --- | --- | --- | --- | --- | --- | --- | --- | --- |
| Age | U | U |  | U | U | U | U | U | U |  |  | U | 9 |
| Sex | U | U |  | U |  |  | U | U | U | U |  |  | 7 |
| Transpired length of stay |  |  |  |  | U | U | U | U | U | U |  | U | 7 |
| Weight |  | U |  | U |  | U |  |  |  | U |  |  | 4 |
| Admission type |  | U |  |  |  | U | U |  |  |  |  |  | 3 |
| Height |  |  |  | U |  | U |  |  |  | U |  |  | 3 |
| Admission source |  | U |  |  |  | U |  |  |  |  |  |  | 2 |
| Care unit type |  |  |  |  |  | U |  |  |  | U |  |  | 2 |
| Had surgery/general anaesthetic flag | U |  |  |  |  |  |  |  |  | U |  |  | 2 |
| Race |  | U |  |  |  |  |  | U |  |  |  |  | 2 |
| Care directive |  |  |  |  |  |  | U |  |  |  |  |  | 1 |
| Comfort care |  |  |  |  |  |  |  |  |  | U |  |  | 1 |
| Season |  |  |  |  |  |  | U |  |  |  |  |  | 1 |
| Time of day |  |  |  |  |  |  | U |  |  |  |  |  | 1 |
| Hours since midnight of the current day |  |  |  |  |  |  |  |  |  |  |  | U | 1 |
| Hospital indicator |  |  |  |  |  |  | U |  |  |  |  |  | 1 |
| Care unit location |  |  |  |  |  |  |  |  |  | U |  |  | 1 |
| Speciality unit |  |  |  |  |  | U |  |  |  |  |  |  | 1 |
| Count of previous ICU stays |  |  |  |  | U |  |  |  |  |  |  |  | 1 |
| Had prior intensive care stay |  |  |  |  |  |  |  |  |  |  |  | U | 1 |

### Table A.4: Calculated variables

| Variable | Bell 2021 (DI) | Brajer 2020 | Kwon et al (DEWS) | Dziadzko 2018 (APPROVE) | Churpek 2014 (eCART) | Kia 2020 (MEWS++) | Kipnis 2016 (AAM) | O'Brien 2019 | Pou-Prom 2022 (CHARTwatch) | Romero-Brufau 2021 (MC-EWS) | Rossetti, 2024 (CONCERN) | Churpek, 2024 (eCARTv5) | Total count |
| --- | --- | --- | --- | --- | --- | --- | --- | --- | --- | --- | --- | --- | --- |
| shock index: hr/sbp |  |  |  | U |  |  | U |  |  | U |  |  | 3 |
| bun creatinine ratio: BUN/Creatinine |  |  |  | U | U |  |  |  |  | U |  |  | 3 |
| body mass index: weight/height^2 |  |  |  | U |  |  |  |  |  |  |  | U | 2 |
| (ag/hco3)x1000 |  |  |  |  |  |  | U |  |  |  |  |  | 1 |
| tlos x laps2 |  |  |  |  |  |  | U |  |  |  |  |  | 1 |
| pulse pressure: systolic-diastolic bp |  |  |  | U |  |  |  |  |  |  |  |  | 1 |
| pulse pressure index |  |  |  |  | U |  |  |  |  |  |  |  | 1 |
| MARS prediction |  |  |  |  |  |  |  |  | U |  |  |  | 1 |
| care setting indicator: not defined |  |  |  |  |  |  |  |  |  | U |  |  | 1 |
| most recent of HR or PR: not defined |  |  |  |  |  |  |  |  |  | U |  |  | 1 |
| most recent of RR or resp. pattern: not defined |  |  |  |  |  |  |  |  |  | U |  |  | 1 |
| Oxygen support binary flag |  |  |  |  |  |  |  |  |  | U |  |  | 1 |
| HR/Hemoglobin ratio |  |  |  |  |  |  |  |  |  | U |  |  | 1 |
| Respiratory index if Oxy support |  |  |  |  |  |  |  |  |  | U |  |  | 1 |
| Respiratory index if no oxy |  |  |  |  |  |  |  |  |  | U |  |  | 1 |
| SpO2-Hemoglobin: spo2*hemoglobin |  |  |  |  |  |  |  |  |  | U |  |  | 1 |
| sp02 * Os support flow |  |  |  |  |  |  |  |  |  | U |  |  | 1 |
| Kirkland probability (see definition) |  |  |  |  |  |  |  |  |  | U |  |  | 1 |

### Table A.5: Treatment variables

| Variable | Bell 2021 (DI) | Brajer 2020 | Kwon et al (DEWS) | Dziadzko 2018 (APPROVE) | Churpek 2014 (eCART) | Kia 2020 (MEWS++) | Kipnis 2016 (AAM) | O'Brien 2019 | Pou-Prom 2022 (CHARTwatch) | Romero-Brufau 2021 (MC-EWS) | Rossetti, 2024 (CONCERN) | Churpek, 2024 (eCARTv5) | Total count |
| --- | --- | --- | --- | --- | --- | --- | --- | --- | --- | --- | --- | --- | --- |
| Total IV fluids | U | U |  |  |  |  |  |  | U |  |  |  | 3 |
| Vasopressor |  | U |  | U |  |  |  |  |  |  |  |  | 2 |
| Antibiotics |  | U |  |  |  |  |  |  |  |  |  |  | 1 |
| Heparain |  | U |  |  |  |  |  |  |  |  |  |  | 1 |
| Opioid |  | U |  |  |  |  |  |  |  |  |  |  | 1 |
| Steroids |  | U |  |  |  |  |  |  |  |  |  |  | 1 |
| Insulin |  | U |  |  |  |  |  |  |  |  |  |  | 1 |
| Benzodiazepines |  | U |  |  |  |  |  |  |  |  |  |  | 1 |
| Immunosuppressants |  | U |  |  |  |  |  |  |  |  |  |  | 1 |
| Chemotherapy |  | U |  |  |  |  |  |  |  |  |  |  | 1 |
| catheter used flag |  |  |  |  |  |  |  |  | U |  |  |  | 1 |
| other output |  |  |  |  |  |  |  |  | U |  |  |  | 1 |
| other intake |  |  |  |  |  |  |  |  | U |  |  |  | 1 |

### Table A.6: Composite indices variables

| Variable | Bell 2021 (DI) | Brajer 2020 | Kwon et al (DEWS) | Dziadzko 2018 (APPROVE) | Churpek 2014 (eCART) | Kia 2020 (MEWS++) | Kipnis 2016 (AAM) | O'Brien 2019 | Pou-Prom 2022 (CHARTwatch) | Romero-Brufau 2021 (MC-EWS) | Rossetti, 2024 (CONCERN) | Churpek, 2024 (eCARTv5) | Total count |
| --- | --- | --- | --- | --- | --- | --- | --- | --- | --- | --- | --- | --- | --- |
| lab-based acute physiology score |  |  |  |  |  |  | U |  |  |  |  |  | 1 |
| Comorbidity point score |  |  |  |  |  |  | U |  |  |  |  |  | 1 |

### Table A.7: Comorbidity flag variables

| Variable | Bell 2021 (DI) | Brajer 2020 | Kwon et al (DEWS) | Dziadzko 2018 (APPROVE) | Churpek 2014 (eCART) | Kia 2020 (MEWS++) | Kipnis 2016 (AAM) | O'Brien 2019 | Pou-Prom 2022 (CHARTwatch) | Romero-Brufau 2021 (MC-EWS) | Rossetti, 2024 (CONCERN) | Churpek, 2024 (eCARTv5) | Total count |
| --- | --- | --- | --- | --- | --- | --- | --- | --- | --- | --- | --- | --- | --- |
| Chronic Kidney Disease |  |  |  |  |  |  |  | U |  |  |  |  | 1 |
| Chronic Obstructive Pulmonary disease |  |  |  |  |  |  |  | U |  |  |  |  | 1 |
| Diabetes |  |  |  |  |  |  |  | U |  |  |  |  | 1 |
| Human Imunodeficiency virus |  |  |  |  |  |  |  | U |  |  |  |  | 1 |
| Malignancy |  |  |  |  |  |  |  | U |  |  |  |  | 1 |
| Miocardial Infarction |  |  |  |  |  |  |  | U |  |  |  |  | 1 |
| Stroke |  |  |  |  |  |  |  | U |  |  |  |  | 1 |
| Transplant |  |  |  |  |  |  |  | U |  |  |  |  | 1 |
